# The Use of Vascular Endothelial Growth Factor Inhibitors and Complementary Treatment Options in Polypoidal Choroidal Vasculopathy: A Subtype of Neovascular Age-Related Macular Degeneration

**DOI:** 10.3390/ijms19092611

**Published:** 2018-09-03

**Authors:** Kelvin Yi Chong Teo, Mark Gillies, Samantha Fraser-Bell

**Affiliations:** 1 Singapore National Eye Centre, Singapore 168751, Singapore; 2 Singapore Eye Research Institute, Singapore 169856, Singapore; 3 Sydney Eye Hospital Foundation, Sydney Eye Hospital, Sydney 2000, Australia; 4 The Save Sight Institute, Sydney Medical School, University of Sydney, Sydney 2000, Australia; mark.gillies@sydney.edu.au (M.G.); sfraserbell@gmail.com (S.F.-B.)

**Keywords:** anti-VEGF, AMD, photodynamic therapy, polypoidal choroidal vasculopathy, PCV

## Abstract

Polypoidal choroidal vasculopathy (PCV) is a subtype of neovascular age-related macular degeneration (AMD; nAMD) which occurs more commonly in Asian populations as compared to Caucasians. PCV and nAMD share pathological mechanisms, including pathological expression of vascular endothelial growth factor (VEGF). The advent of anti-vascular endothelial growth factor (VEGF) revolutionized the treatment of nAMD. Despite being a subtype of nAMD, PCV responds less well to VEGF inhibitors; thus, photodynamic therapy (PDT) in combination with anti-VEGF treatment may be considered. This review aims to summarize the current evidence for the treatment of PCV, especially whether VEGF inhibitors should be used alone or in combination with PDT.

## 1. Introduction

Age-related macular degeneration (AMD) is one of the leading causes of blindness worldwide in people over the age of 50 [1]. By 2040, it is estimated that 288 million people will have AMD with the largest proportion from Asia (113 million) [2]. Early AMD, which is often asymptomatic, is characterized by typical clinical signs such as the presence of drusen and pigmentary abnormalities [3,4]. Advanced AMD, which can be divided into non-neovascular and neovascular AMD (nAMD), has a guarded visual prognosis. Advanced non-neovascular AMD is characterized by geographic or macular atrophy which causes loss of central vision if it involves the center of the fovea. Neovascular AMD is characterized by the presence of a choroidal neovascular network (CNV) which results in subretinal exudation and rapid loss of vision [4]. Intravitreal administration of anti-vascular endothelial growth factor (anti-VEGF) agents revolutionized the treatment of nAMD by not only stabilizing vision, but also improving it in many cases [1,5,6].

Polypoidal choroidal neovascularization (PCV) is considered a subtype of nAMD and is characterized by polypoidal lesions arising from terminal ends of branching vascular networks which are best diagnosed on indocyanine green angiography (ICGA) [7]. More than half (50–80%) of large serosanguinous maculopathy in nAMD can be attributed to PCV [8,9], which may also be accompanied by extensive sub-retinal or sub-retinal pigment epithelium (RPE) hemorrhage, often resulting in acute and severe vision loss. PCV was reported to be more common in Asians [10,11,12,13,14] than in Caucasians [15,16,17,18]. While anti-VEGF therapy is now the standard of care for nAMD, there is varying evidence regarding the efficacy of anti-VEGF treatment in PCV [14]. 

This article aims to summarize the clinical characteristics, epidemiology, and best evidence to date for the treatment of PCV. PubMed (https://www.ncbi.nlm.nih.gov/pubmed), EMBASE (www.elsevier.com/embase), Web of Science (www.webofknowledge.com/), Medline (https://www.nlm.nih.gov/bsd/medline.html), and Cochrane Library (https://www.cochranelibrary.com/) were searched for relevant studies on PCV. The following terms were used and adapted for the searches in each database: (polypoidal choroidal vasculopathy or PCV) and (vascular endothelial growth factors or anti-VEGF or angiogenesis inhibitors or ranibizumab or lucentis or bevacizumab or avastin or aflibercept) and (photodynamic therapy or PDT). When titles and/or abstracts fit the index words, the full article was retrieved.

## 2. Epidemiology of PCV

The accurate estimation of PCV prevalence is difficult since the diagnosis of PCV cannot be made simply through clinical examination and standard imaging techniques, such as optical coherence tomography (OCT) and fluorescein angiography (FA), in many cases. The gold-standard imaging modality for diagnosing PCV is ICGA which is not routinely performed in many clinical practices [19]. Most epidemiological studies on PCV report a higher prevalence in pigmented races like Asian (22–62% of AMD) than in the Caucasian population (5–20% of AMD) [16,20,21,22,23,24,25,26,27].

## 3. Risk Factors

Typical nAMD and PCV share many risk factors despite affecting different populations. Cigarette smoking was reported as a risk factor in the development of both conditions (odds ratios 4.4–4.8 for PCV and 4.9 for typical AMD) [28], as was a higher body mass index, male gender, hyperlipidemia, and hypertension [28,29,30]. Typical nAMD and PCV share some genetic associations, such as the polymorphisms of the complement factor H gene (*CFH*) Y402H, and *ARMS2* A69S [31]. Specific genetic associations were shown to exist for PCV, such as a variant of the cholesteryl ester transfer protein (CETP) locus, which was shown to be associated with an increased risk of PCV [32,33].

## 4. Clinical Features of PCV

**Figure 1:**
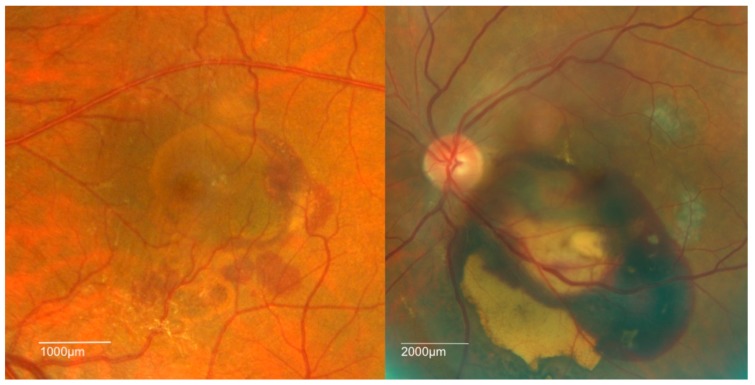
Fundus photographs showing the two clinical patterns of polypoidal choroidal vasculopathy (PCV): hemorrhagic (**right**) and serous (**left**).

**Figure 2:**
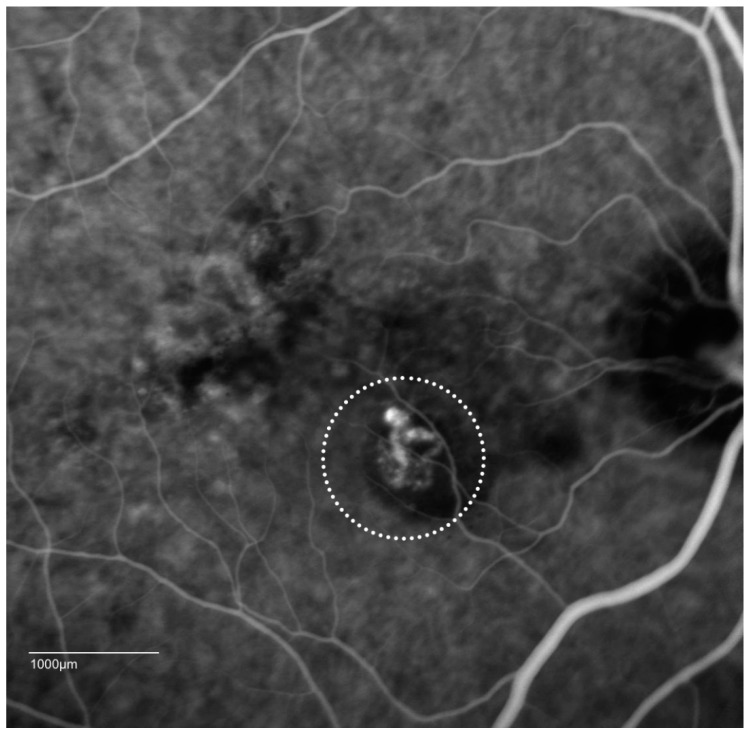
Indocyanine green angiographic (ICGA) patterns of PCV: cluster of grapes configuration (dotted circle) with a hypofluorescent halo. Video angiography sometimes shows pulsatile lesions, which is a definitive sign for the diagnosis of PCV.

Polypoidal choroidal vasculopathy is characterized clinically by the presence of polypoidal lesions which are sometimes visible on fundoscopy as orange-red nodules. These polyps or nodules are often associated with serosanguinous pigment epithelial detachments (PED) without associated drusen. A notch in the margin of a PED often indicates the site of the polyps. The PED can be serous or hemorrhagic in nature with the hemorrhagic type carrying a worse visual prognosis [7,17,34,35]. (Figure 1) The diagnosis of PCV is often challenging and must be confirmed with ICGA (Figure 2) which allows for better visualization of the choroidal vasculature than fundus fluorescein angiography. This is because its higher protein-binding affinity prevents it leaking from the normal choriocapillaris as fluorescein does. In addition, indocyanine green emits near-infrared light, which penetrates the RPE more readily than the green light emitted by fluorescein. The diagnosis of PCV on ICGA requires the presence of focal hyperfluorescence with the presence of at least one of the following: an associated branching vascular network (BVN), pulsatile polyp, nodular appearance on stereoscopic viewing, hypofluorescent halo, orange subretinal nodule, or presence of massive submacular hemorrhage on clinical exam [36].

**Figure 3:**
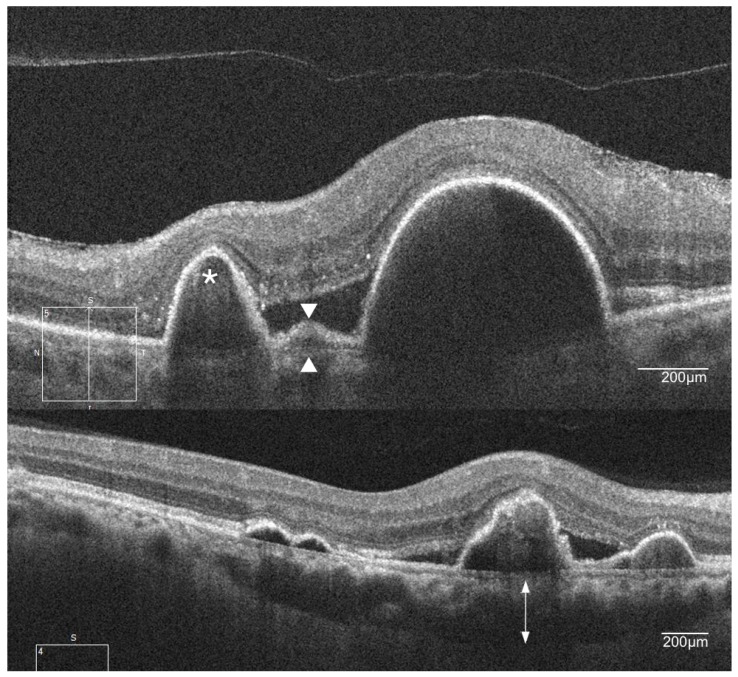
Spectral domain optical coherence tomography (OCT) features of PCV. The image on the top shows a sharp elevation of the retinal pigment epithelium (RPE) with underlying moderate reflectivity (asterisk) and double-layer sign, consisting of two hyper-reflective lines (white arrowheads). The bottom image shows the underlying thickened choroid (double-headed arrow).

Several reports suggested a high correlation between the presence of specific optical coherence tomography (OCT) features and PCV [37,38,39]. Various features such as a peaked PED with a notch, and a double layer consisting of two hyper-reflective lines representing Bruch’s membrane and RPE separated by the BVN are highly suggestive of PCV [37,38,39] (Figure 3).

The presence of a thick choroid (pachychoroid) in PCV led to the suggestion that PCV falls within the pachychoroid spectrum of conditions that may have a different cause from typical nAMD. Focal changes in the choroid appear to correspond to the areas where PCV lesions manifest [40]. The abnormal vascularization in PCV (polyps and BVN) which is present between the RPE and the outer portion of Bruch’s membrane is consistent with “type 1” neovascularization in typical nAMD [41]. The difference, however, is that enhanced depth imaging OCT reveals a thickened choroidal layer in eyes with PCV rather than the choroidal thinning that often is observed in eyes with type 1 lesions in typical nAMD [42,43,44] (Figure 3).

## 5. Overview of Anti-VEGF Treatment of nAMD

VEGF-A is a potent stimulator of vascular endothelial cell growth which is required in angiogenesis, leucocyte recruitment, and vessel permeability. VEGF is thought to have a key role in the proliferation of pathologic neovascularization in retinal angiogenic diseases such as nAMD, diabetic retinopathy (DR), and retinal vein occlusion (RVO) [45,46].

There are several anti-VEGF agents commonly used in nAMD. The first agent approved for use was pegatanib (Macugen, Eyetech Inc., Palm Beach Gardens, FL, USA) [47]; however, ranibizumab (Lucentis, Genentech, CA, USA/Novartis AG, Basel, Switzerland), aflibercept (Eylea, Regeneron, Tarrytown, NY, USA/Bayer Healthcare, Berlin, Germany), and the off-label use of bevacizumab (Avastin, Genentech, CA, USA/Roche, Basel, Switzerland) have since superseded pegaptanib, as they were shown to have much better outcomes [48,49,50].

Ranibizumab is a 48-kDa antibody fragment that targets all isoforms of VEGF, while aflibercept is a fusion protein (115 kDa) consisting of VEGF-binding portions from the extracellular domains of VEGFR-1 and -2 fused to human immunoglobulin G (IgG) that binds VEGF-A and placental growth factor (PlGF). Bevacizumab is a full-length humanized monoclonal antibody that binds to all isoforms of VEGF. It is approved only as an intravenous therapy for systemic malignancies, but is used off-label for nAMD and other retinal angiogenic diseases with good effect.

Multiple landmark clinical trials demonstrated the efficacy and safety of the use of anti-VEGF for the treatment of nAMD [48,51]. The pivotal Minimally Classic/Occult Trial of the Anti-VEGF Antibody Ranibizumab in the Treatment of Neovascular Age-Related Macular Degeneration (MARINA) and Anti-VEGF Antibody for the Treatment of Predominantly Classic Choroidal Neovascularization in Age-Related Macular Degeneration (ANCHOR) trials showed an improvement in vision by 7–11 letters with monthly ranibizumab injections over 12 months compared with previous laser-based therapies [48]. The VEGF Trap-Eye: Investigation of Efficacy and Safety in Wet AMD (VIEW) studies, which assessed aflibercept, reported non-inferior outcomes in eyes treated with three initial monthly injections and subsequent two-monthly injections compared with monthly dosing of ranibizumab 0.5 mg [52,53]. Bevacizumab was found to be generally non-inferior to ranibizumab when used with the same dosing regimen in the Comparison of Age-Related Macular Degeneration Treatment Trial (CATT), and the Inhibit VEGF in Age-related choroidal Neovascularisation (IVAN) and Groupe d’Evaluation Français Avastin versus Lucentis (GEFAL) trials [54,55].

## 6. Anti-VEGF Monotherapy in PCV

Biomarker studies that examined the level of VEGF in the aqueous humor of eyes with PCV reported higher levels of VEGF than in controls, but lower than that of eyes with typical nAMD [56,57]. The evidence for the treatment of PCV with anti-VEGF therapy is less extensive than for typical nAMD as PCV was often not specifically identified in many of the AMD trials. Nonetheless, early studies reported the stabilization of vision in PCV with the use of both bevacizumab and ranibizumab, but no anatomical resolution of the lesions [58,59]. Later studies with longer follow-ups and larger cohorts reported 17–40% of eyes achieving more than 15-letter improvement after 12 months of treatment, with polyp closure in around 25% at six months and 40% and 24% at 12 and 24 months, respectively [60,61,62,63,64]. Many of these trials followed a treatment regimen of an initial induction phase of monthly injections over two months followed by an as-needed or pro re nata (PRN) reinjection protocol of ranibizumab. Smaller lesion size, the absence of PED at baseline, and no CNV recurrences were found to predict better vision outcomes [65]. The Polypoidal Choroidal Vasculopathy with Intravitreal Ranibizumab (PEARL) studies reported results of ranibizumab at different concentrations for the treatment of PCV (0.5 mg for 12 months in PEARL 1, and 2 mg for six months in PEARL 2) following a strict monthly injection regimen [62,66]. The proportion of patients who gained 15 or more letters was similar in PEARL 1 and PEARL 2 (23% and 26%) with no patients losing more than 15 letters. More polypoidal lesions were resolved in PEARL 2 than in PEARL 1 (79% compared to 38%).

The Ranibizumab (Lucentis) And Photodynamic Therapy On Polypoidal choroidal vasculopathy (LAPTOP) study was a phase-4 prospective, multicenter, randomized trial comparing the effect of initial PDT versus ranibizumab in the treatment of PCV using a PRN treatment protocol. Patients achieved better visual outcomes in the ranibizumab arm compared to the PDT arm at both month 12 and month 24. Angiographic results, however, were not evaluated in these studies; thus, polyp closure rate could not be determined [63].

Another phase-4 randomized, double-masked, multicenter trial based in China, the Efficacy and Safety of Ranibizumab 0.5 mg Administered as Two Alternative Dosing Regimens in Chinese Patients With nAMD (DRAGON) study, compared the efficacy of ranibizumab monotherapy using a monthly fixed dosing regimen versus a PRN regimen in patients with nAMD. A subset analysis from baseline ICGA images reported that 41.7% of the 334 enrolled patients had PCV. There was significant improvement in vision in both PCV and non-PCV eyes in the monthly fixed dosing arm (+12.7 letters and +11.2 letters, respectively) and the PRN arm (+9.4 letters and +8.4 letters, respectively) at 24 months [67].

Several studies, including the Efficacy of fixed-dosing aflibercept for treating polypoidal choroidal vasculopathy (VAULT) study, examined the use of aflibercept monotherapy in the treatment of PCV and reported favorable visual outcomes (5–10 letter gains) and polyp regression rates (65–69%) over 12 months [68,69,70,71]. In a prospective study of 21 eyes where aflibercept was administered monthly for three initial treatments then every other month, vision was reported to stabilize at six months with a 67% polyp closure rate [72]. Another study reported no difference in the improvement of both functional and anatomical outcomes between fixed bimonthly dosing and a PRN regimen after three initial monthly injections of aflibercept [73]. A post-hoc analysis of the VIEW studies, a series of phase-3 trials comparing two-month dosing of aflibercept with monthly ranibizumab in eyes with nAMD, showed no difference in visual outcomes or reduction in retinal thickness between eyes with PCV and no PCV when treated with aflibercept. Unfortunately, no post-treatment ICGA was available to determine the closure rate of the polyps [74].

There are fewer studies reporting the outcomes of bevacizumab monotherapy on PCV, and most of them had short follow-ups or were not performed on treatment-naive patients. Most were retrospective in nature and did not adhere to a fixed dosing regimen [58,75,76,77]. 

## 7. Application of the Photosensitizer Verteporfin in Photodynamic Therapy

Photodynamic therapy (PDT) utilizes the photosensitive verteporfin in combination with an infrared laser to induce the regression of the CNV in nAMD. The exact mechanism of action for PDT is unknown, with animal studies reporting that PDT induces endothelial cell destruction, clot formation, and vascular occlusion of the choroidal neovascular complex with minimal damage to adjacent retinal structures [78,79]. Before the advent of anti-VEGF therapy, PDT proved to be efficacious in the closure of classic type nAMD; however, gains in vision were limited [80,81]. It was also widely used in the treatment of PCV [82,83,84,85]; however, visual outcomes beyond 12 months were disappointing, and vision eventually returned to baseline after three years [86]. Complications, including choroidal infarction, RPE tears, and subretinal hemorrhage, while rare, further limited the use of PDT, especially in eyes with good presenting vision [87,88,89]. Several modifications to the PDT settings were proposed that appear to reduce the rate of these complications, such as reduced-fluence PDT and limiting the spot size to only treat active polyps [90]. With reports on the increasing efficacy of anti-VEGF therapy, however, PDT monotherapy largely fell out of favor as a treatment for PCV.

## 8. Landmark Trials and PDT Combination Therapy

Two recent landmark randomized controlled trials (Efficacy and safety of verteporfin photodynamic therapy in combination with ranibizumab or alone versus ranibizumab monotherapy in patients with symptomatic macular polypoidal choroidal vasculopathy (EVEREST II) and Aflibercept in polypoidal choroidal vasculopathy (PLANET)) reported the results of anti-VEGF therapy and PDT in combination for the treatment of PCV. The trials differed in the timing of PDT administration. The EVEREST I study compared the rate of polyp regression at six months among the three treatment arms in 61 subjects: PDT monotherapy, PDT in combination with ranibizumab 0.5 mg, and ranibizumab monotherapy. The study reported that PDT alone or combined with ranibizumab achieved a significantly higher polyp regression rate (71.4% and 77.8%) compared with ranibizumab alone (28.6%). Ranibizumab monotherapy, however, achieved higher visual gains than PDT monotherapy (+9.2 letters versus +7.5 letters), although the difference was not statistically significant and the study was not powered to examine vision change as the primary outcome [36]. The larger EVEREST II study was a trial of 322 Asian participants with PCV, which compared the efficacy of ranibizumab monotherapy with PDT/ranibizumab combination therapy. Eyes in the ranibizumab monotherapy arm gained an average of in 5.1 letters with a polyp closure rate of 34.7% compared to eyes in the combination arm which gained 8.3 letters with a polyp closure rate of 69.3% at 12 months. A mean of seven injections was administered over 12 months following a regimen of PRN treatments after three initial monthly doses in the monotherapy group as compared to four injections in the combination group following the same treatment regimen [91]. 

The Initial versus delayed photodynamic therapy in combination with ranibizumab for treatment of polypoidal choroidal vasculopathy (FUJISAN) study evaluated the outcomes of initial or deferred PDT combined with ranibizumab in 72 patients. Patients in the deferred PDT arm were evaluated after three initial monthly ranibizumab injections, and deferred PDT was administered if the re-treatment criteria were met. Similar vision and polyp closure outcomes were reported in both arms at one year. More than half the patients in the deferred arm did not require PDT over 12 months; however, they had significantly more injections (3.8 vs. 1.5 injections in addition to three loading doses) [92].

The PLANET study assessed the efficacy of aflibercept in the treatment of PCV. This study used fixed dosing of aflibercept with and without rescue PDT, which was available after three months. Both treatment arms achieved similar vision gains (10.7 versus 10.9 letters), but importantly, the majority of eyes (>90%) did not meet rescue criteria, meaning that the majority received aflibercept alone. Polyp regression rates were also similar in both arms (38.9% vs. 44.8%) [93].

Both the EVEREST II and PLANET studies reported significant visual acuity gain in the anti-VEGF monotherapy arms at one year (5.1 letters in EVEREST II and 10.8 letters in PLANET) with similar polyp closure rates (34.7% in EVEREST II and 38.9% in PLANET). Anti-VEGF treatments administered were also similar with 7.3 in the EVERESTII and 8.1 in PLANET. EVEREST II, however, reported 51% of eyes with no disease activity (defined as absence of persistent or new polyps based on OCT, FA, ICGA, and color fundus examinations) at 12 months, whereas PLANET reported 81.7% of eyes with no active polyps (active polyps defined as polyps with leakage on FA, subretinal or intra-retinal fluid on OCT, or presence of new hemorrhage).

The EVEREST II concluded that combination therapy achieved superior vision gains and polyp closure rates with fewer ranibizumab injections than monotherapy in the first year of treatment. In contrast, the PLANET study concluded that aflibercept monotherapy achieved significant vision gains in more than 85% of the patients with no added benefit from combination therapy with PDT at one year.

Based on these two landmark studies, anti-VEGF monotherapy with either ranibizumab or aflibercept can achieve visual improvement and reduction in disease activity in patients with PCV. With no head-to-head studies comparing anti-VEGF agents in the treatment of PCV, it is tempting to compare the letters gained between different agents from the monotherapy arms of the two trials (+10.8 letters with the administration of aflibercept in PLANET compared with +5.1 letters with the administration of ranibizumab in EVEREST II). This should be done with caution as both trials had participants with different baseline vision, and generally, eyes with lower baseline vision (as in PLANET) can be expected to achieve a larger magnitude of improvement [94]. Differences in dosing regimens between the two trials should also be considered as a potential factor underlying any differences in absolute letter change reported. The various randomized controlled trials for the treatment of PCV are summarized in Table 1.

We reported similar findings to the EVEREST II trial in a real-world observational study of a cohort of 193 eyes diagnosed with PCV. The combination group (PDT and anti-VEGF) gained more letters (14.3 letters) at 12 months than those receiving monotherapy (8.4 letters). Our higher letter gain compared to that found in the EVEREST II trial may be due to a lower starting baseline visual acuity. We also reported a lower mean number of treatments (4.3 versus 6.4 injections) and shorter time to inactive disease (80 days versus 150 days) in the combination group compared to the monotherapy group.

Eyes with PCV that were treated with either bevacizumab or ranibizumab were reported to have similar visual outcomes, polyp regression rates, central macular thickness, and mean number of injections by a six-month retrospective study of 126 eyes [95]. Similarly, we did not find different visual or anatomical outcomes from different anti-VEGF agents in our real-world cohort study; however, many fewer eyes received either ranibizumab (*n* = 13) or aflibercept (*n* = 16) than bevacizumab (*n* = 83).

## 9. Current Treatment Recommendations for PCV

Treatment of PCV, as for typical nAMD, should focus on achieving the best possible visual outcome with minimal treatment burden. Considering both landmark trials, the EVEREST II and PLANET studies, anti-VEGF monotherapy, regardless of type, and combination therapy with PDT appear to provide excellent visual outcomes at 12 months. Both approaches appear to be acceptable; hence, when considering which treatment modality to administer, one must consider the individual patient’s needs and healthcare jurisdictions. It may be difficult to administer PDT in some healthcare settings due to poor access to verteporfin or the PDT laser, while, in other places, there may be a much higher financial burden when using on-label anti-VEGF monotherapy. The advantage of anti-VEGF monotherapy is the elimination of the need of ICGA, as PCV can be treated like typical nAMD without the need for differentiation. The disadvantages is that the number of treatments with monotherapy can be expected to be much higher than with combination therapy; thus, patients will need to commit to more frequent follow-ups and multiple injections. Repeated injections also increase the cumulative risk of complications like endophthalmitis, which, while rare, can have devastating effects on vision. The advantage of combination therapy with PDT and anti-VEGF treatment is the lower eventual treatment burden with anti-VEGF therapy. PDT, on the other hand, also has serious complications which are not rare, such as choroidal infarction, subretinal hemorrhage, and RPE rips, which can result in irreversible vision loss. Several imaging features of PCV lesions, such as choroidal thickness, lesion size, and polyp configuration, were suggested to help predict response to combination therapy; however, these factors remain to be investigated in more detail in larger controlled clinical studies [96].

A gap in our knowledge regarding the management of PCV is the importance of polyp closure. In these current studies, polyp closure rates did not seem to affect final vision outcome, although one might intuitively expect that polyp closure is a marker for stability. In addition, longer-term studies over 3–5 years also did not report any association between polyp closure and recurrence rates [65,97]. With the current level-1 evidence reporting only outcomes at 12 months, there is insufficient long-term evidence to determine the association between recurrence rate and long-term visual outcomes.

In conclusion, PCV can be effectively treated with both combination PDT with anti-VEGF therapy and anti VEFG monotherapy. The treatment option, therefore, depends on the individual patient and healthcare setting. In patients with poor access to PDT, anti-VEGF monotherapy may be the treatment option of choice, while combination therapy may be ideal for patients that cannot commit to regular follow-ups and multiple treatments.

## Figures and Tables

**Table 1:** Summary of randomized controlled trials in the treatment of polypoidal choroidal vasculopathy (PCV).

Study	Follow-Up, Months	Treatment	Sample Size	Number of Injections	Number of PDT	Polyp Regression Rate, %	Baseline Vision, Letters	Mean Vision Change, Letters
LAPTOP (2014)	12	Ranibizumab 3 × monthly + PRN	47	5.8	-	Not reported	88.0	4.0
		PDT	46	5.2	1.5	Not reported	84.0	−2.0
FUJISAN (2015)	12	Initial PDT + ranibizumab 3 × monthly + PRN	37	4.5	1.1	62.1	54.3	8.1
		Ranibizumab 3 × monthly + PRN + deferred PRN PDT	35	6.8	1.4	54.8	54.9	8.8
EVEREST I (2012)	6	Ranibizumab 3 × monthly + PRN	21	5.2	1.9 (sham)	28.6	49.0	9.2
		PDT + ranibizumab PRN	19	3.9	1.7	77.8	57.2	10.9
		PDT	21	4.2	1.4	71.4	56.6	7.5
EVEREST II (2017)	12	Ranibizumab 3 × monthly + PRN	168	7.3	2.3 (sham)	34.7	61.1	5.1
		PDT + ranibizumab PRN	154	5.2	1.5	69.3	61.2	8.3
PLANET (2017)	12	Aflibercept 3 × monthly + 8-weekly	318	8.1	-	38.9	57.7	10.7
		Aflibercept 3 × monthly + 8-weekly + rescue PDT		8.0	0.2	44.8	59.0	10.9
LAPTOP (2014)	12	Ranibizumab 3 × monthly + PRN	47	5.8	-	Not reported	88.0	4.0
		PDT	46	5.2	1.5	Not reported	84.0	−2.0
FUJISAN (2015)	12	Initial PDT + ranibizumab 3 × monthly + PRN	37	4.5	1.1	62.1	54.3	8.1
		Ranibizumab 3 × monthly + PRN + deferred PRN PDT	35	6.8	1.4	54.8	54.9	8.8
EVEREST I (2012)	6	Ranibizumab 3 × monthly + PRN	21	5.2	1.9 (sham)	28.6	49.0	9.2
		PDT + ranibizumab PRN	19	3.9	1.7	77.8	57.2	10.9
		PDT	21	4.2	1.4	71.4	56.6	7.5
EVEREST II (2017)	12	Ranibizumab 3 × monthly + PRN	168	7.3	2.3 (sham)	34.7	61.1	5.1
		PDT + ranibizumab PRN	154	5.2	1.5	69.0	61.2	8.3
PLANET (2017)	12	Aflibercept 3 × monthly + 8-weekly	318	8.1	-	38.9	57.7	10.7
		Aflibercept 3 × monthly + 8-weekly + rescue PDT		8.0	0.2	44.8	59.0	10.9

LAPTOP: Ranibizumab (Lucentis) And Photodynamic Therapy On Polypoidal choroidal vasculopathy study; FUJISAN: Initial versus delayed photodynamic therapy in combination with ranibizumab for treatment of polypoidal choroidal vasculopathy study; EVEREST: Efficacy and safety of verteporfin photodynamic therapy in combination with ranibizumab or alone versus ranibizumab monotherapy in patients with symptomatic macular polypoidal choroidal vasculopathy study; PLANET: Aflibercept in polypoidal choroidal vasculopathy study.
